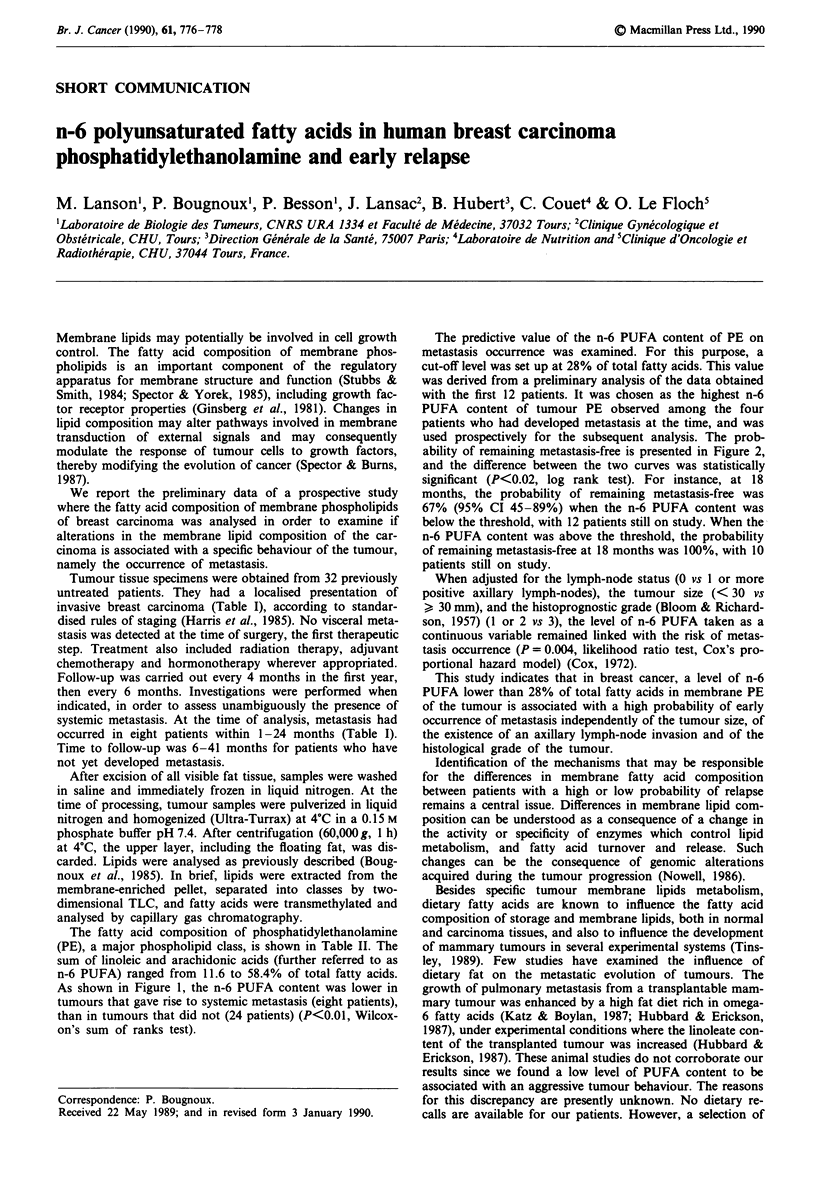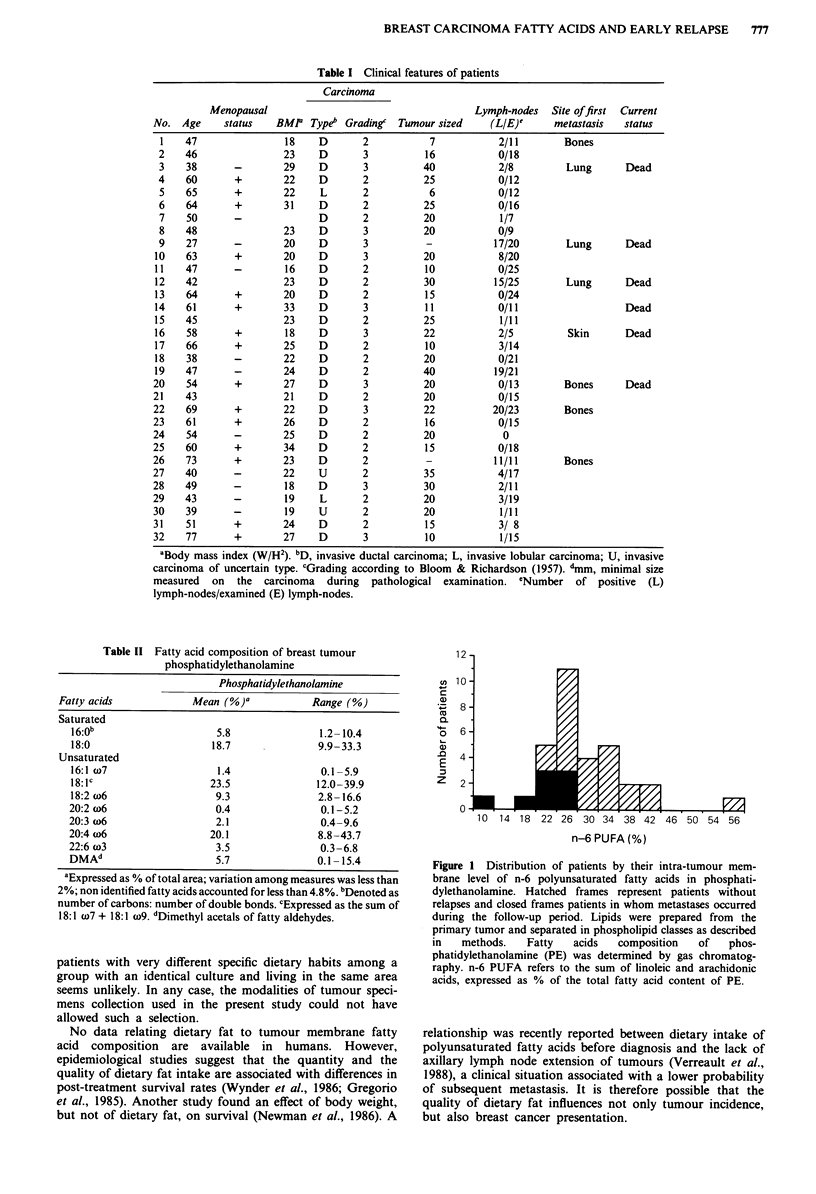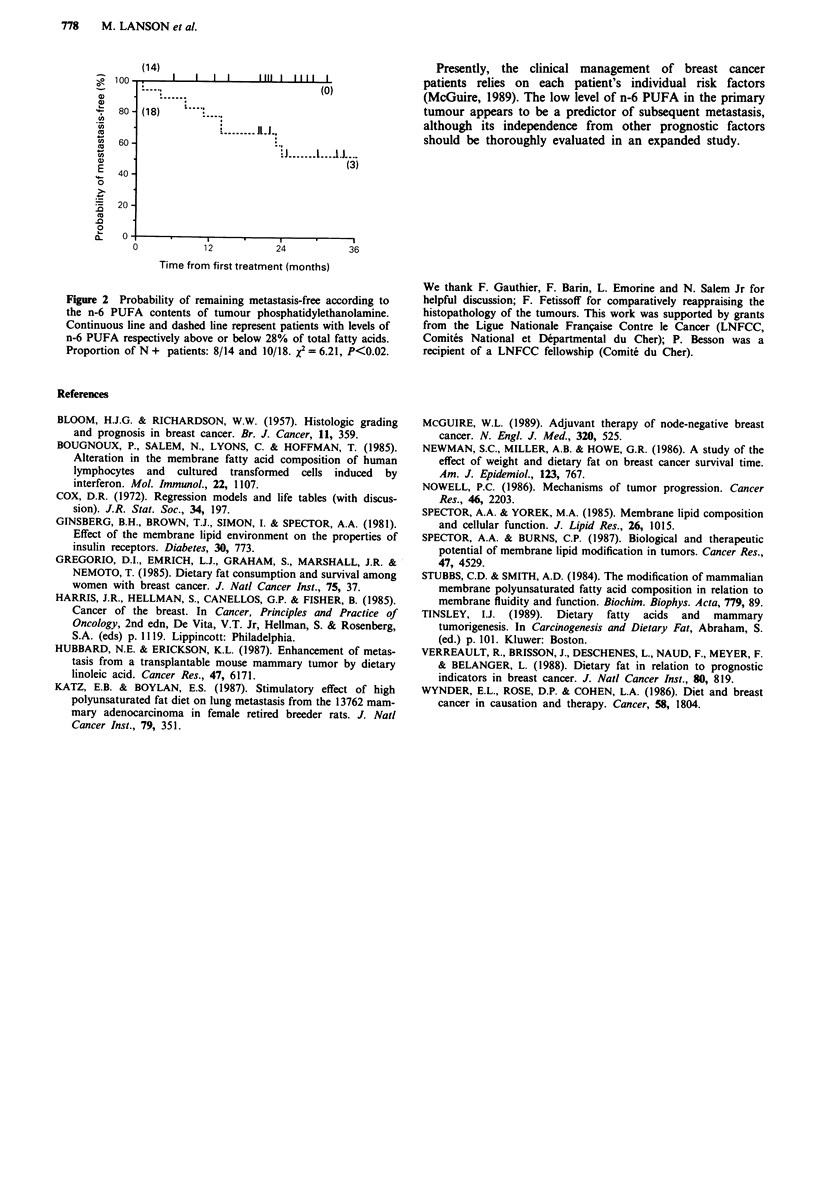# N-6 polyunsaturated fatty acids in human breast carcinoma phosphatidylethanolamine and early relapse.

**DOI:** 10.1038/bjc.1990.174

**Published:** 1990-05

**Authors:** M. Lanson, P. Bougnoux, P. Besson, J. Lansac, B. Hubert, C. Couet, O. Le Floch

**Affiliations:** Laboratoire de Biologie des Tumeurs, CNRS URA 1334 et Faculté de Médecine, Tours, France.


*Br. J. Cancer* (1990), **61**, 776–778 © Macmillan Press Ltd., 1990

SHORT COMMUNICATION

### n-6 polyunsaturated fatty acids in human breast carcinoma phosphatidylethanolamine and early relapse

M. Lanson[1], P. Bougnoux[1], P. Besson[1], J. Lansac[2], B. Hubert[3], C. Couet[4] & O. Le Floch[5]

[1]*Laboratoire de Biologie des Tumeurs, CNRS URA 1334 et Faculté de Médecine, 37032 Tours;* [2]*Clinique Gynécologique et Obstétricale, CHU, Tours;* [3]*Direction Générale de la Santé, 75007 Paris;* [4]*Laboratoire de Nutrition and* [5]*Clinique d'Oncologie et Radiothérapie, CHU, 37044 Tours, France.*

Membrane lipids may potentially be involved in cell growth control. The fatty acid composition of membrane phospholipids is an important component of the regulatory apparatus for membrane structure and function (Stubbs & Smith, 1984; Spector & Yorek, 1985), including growth factor receptor properties (Ginsberg *et al.*, 1981). Changes in lipid composition may alter pathways involved in membrane transduction of external signals and may consequently modulate the response of tumour cells to growth factors, thereby modifying the evolution of cancer (Spector & Burns, 1987).

We report the preliminary data of a prospective study where the fatty acid composition of membrane phospholipids of breast carcinoma was analysed in order to examine if alterations in the membrane lipid composition of the carcinoma is associated with a specific behaviour of the tumour, namely the occurrence of metastasis.

Tumour tissue specimens were obtained from 32 previously untreated patients. They had a localised presentation of invasive breast carcinoma (Table I), according to standardised rules of staging (Harris *et al.*, 1985). No visceral metastasis was detected at the time of surgery, the first therapeutic step. Treatment also included radiation therapy, adjuvant chemotherapy and hormonotherapy wherever appropriated. Follow-up was carried out every 4 months in the first year, then every 6 months. Investigations were performed when indicated, in order to assess unambiguously the presence of systemic metastasis. At the time of analysis, metastasis had occurred in eight patients within 1–24 months (Table I). Time to follow-up was 6–41 months for patients who have not yet developed metastasis.

After excision of all visible fat tissue, samples were washed in saline and immediately frozen in liquid nitrogen. At the time of processing, tumour samples were pulverized in liquid nitrogen and homogenized (Ultra-Turrax) at 4°C in a 0.15 M phosphate buffer pH 7.4. After centrifugation (60,000 *g*, 1 h) at 4°C, the upper layer, including the floating fat, was discarded. Lipids were analysed as previously described (Bougnoux *et al.*, 1985). In brief, lipids were extracted from the membrane-enriched pellet, separated into classes by two-dimensional TLC, and fatty acids were transmethylated and analysed by capillary gas chromatography.

The fatty acid composition of phosphatidylethanolamine (PE), a major phospholipid class, is shown in Table II. The sum of linoleic and arachidonic acids (further referred to as n-6 PUFA) ranged from 11.6 to 58.4% of total fatty acids. As shown in Figure 1, the n-6 PUFA content was lower in tumours that gave rise to systemic metastasis (eight patients), than in tumours that did not (24 patients) ($P<0.01$, Wilcoxon's sum of ranks test).

Correspondence: P. Bougnoux.
Received 22 May 1989; and in revised form 3 January 1990.

The predictive value of the n-6 PUFA content of PE on metastasis occurrence was examined. For this purpose, a cut-off level was set up at 28% of total fatty acids. This value was derived from a preliminary analysis of the data obtained with the first 12 patients. It was chosen as the highest n-6 PUFA content of tumour PE observed among the four patients who had developed metastasis at the time, and was used prospectively for the subsequent analysis. The probability of remaining metastasis-free is presented in Figure 2, and the difference between the two curves was statistically significant ($P<0.02$, log rank test). For instance, at 18 months, the probability of remaining metastasis-free was 67% (95% CI 45–89%) when the n-6 PUFA content was below the threshold, with 12 patients still on study. When the n-6 PUFA content was above the threshold, the probability of remaining metastasis-free at 18 months was 100%, with 10 patients still on study.

When adjusted for the lymph-node status (0 *vs* 1 or more positive axillary lymph-nodes), the tumour size ($<30$ *vs* $\geqslant 30$ mm), and the histoprognostic grade (Bloom & Richardson, 1957) (1 or 2 *vs* 3), the level of n-6 PUFA taken as a continuous variable remained linked with the risk of metastasis occurrence ($P = 0.004$, likelihood ratio test, Cox's proportional hazard model) (Cox, 1972).

This study indicates that in breast cancer, a level of n-6 PUFA lower than 28% of total fatty acids in membrane PE of the tumour is associated with a high probability of early occurrence of metastasis independently of the tumour size, of the existence of an axillary lymph-node invasion and of the histological grade of the tumour.

Identification of the mechanisms that may be responsible for the differences in membrane fatty acid composition between patients with a high or low probability of relapse remains a central issue. Differences in membrane lipid composition can be understood as a consequence of a change in the activity or specificity of enzymes which control lipid metabolism, and fatty acid turnover and release. Such changes can be the consequence of genomic alterations acquired during the tumour progression (Nowell, 1986).

Besides specific tumour membrane lipids metabolism, dietary fatty acids are known to influence the fatty acid composition of storage and membrane lipids, both in normal and carcinoma tissues, and also to influence the development of mammary tumours in several experimental systems (Tinsley, 1989). Few studies have examined the influence of dietary fat on the metastatic evolution of tumours. The growth of pulmonary metastasis from a transplantable mammary tumour was enhanced by a high fat diet rich in omega-6 fatty acids (Katz & Boylan, 1987; Hubbard & Erickson, 1987), under experimental conditions where the linoleate content of the transplanted tumour was increased (Hubbard & Erickson, 1987). These animal studies do not corroborate our results since we found a low level of PUFA content to be associated with an aggressive tumour behaviour. The reasons for this discrepancy are presently unknown. No dietary recalls are available for our patients. However, a selection of

**Table I** Clinical features of patients

| | | | | Carcinoma | | | | | |
|---|---|---|---|---|---|---|---|---|---|
| *No.* | *Age* | *Menopausal status* | *BMI*[a] | *Type*[b] | *Grading*[c] | *Tumour size*[d] | *Lymph-nodes (L/E)*[e] | *Site of first metastasis* | *Current status* |
| 1 | 47 | | 18 | D | 2 | 7 | 2/11 | Bones | |
| 2 | 46 | | 23 | D | 3 | 16 | 0/18 | | |
| 3 | 38 | – | 29 | D | 3 | 40 | 2/8 | Lung | Dead |
| 4 | 60 | + | 22 | D | 2 | 25 | 0/12 | | |
| 5 | 65 | + | 22 | L | 2 | 6 | 0/12 | | |
| 6 | 64 | + | 31 | D | 2 | 25 | 0/16 | | |
| 7 | 50 | – | | D | 2 | 20 | 1/7 | | |
| 8 | 48 | | 23 | D | 3 | 20 | 0/9 | | |
| 9 | 27 | – | 20 | D | 3 | – | 17/20 | Lung | Dead |
| 10 | 63 | + | 20 | D | 3 | 20 | 8/20 | | |
| 11 | 47 | – | 16 | D | 2 | 10 | 0/25 | | |
| 12 | 42 | | 23 | D | 2 | 30 | 15/25 | Lung | Dead |
| 13 | 64 | + | 20 | D | 2 | 15 | 0/24 | | |
| 14 | 61 | + | 33 | D | 3 | 11 | 0/11 | | Dead |
| 15 | 45 | | 23 | D | 2 | 25 | 1/11 | | |
| 16 | 58 | + | 18 | D | 3 | 22 | 2/5 | Skin | Dead |
| 17 | 66 | + | 25 | D | 2 | 10 | 3/14 | | |
| 18 | 38 | – | 22 | D | 2 | 20 | 0/21 | | |
| 19 | 47 | – | 24 | D | 2 | 40 | 19/21 | | |
| 20 | 54 | + | 27 | D | 3 | 20 | 0/13 | Bones | Dead |
| 21 | 43 | | 21 | D | 2 | 20 | 0/15 | | |
| 22 | 69 | + | 22 | D | 3 | 22 | 20/23 | Bones | |
| 23 | 61 | + | 26 | D | 2 | 16 | 0/15 | | |
| 24 | 54 | – | 25 | D | 2 | 20 | 0 | | |
| 25 | 60 | + | 34 | D | 2 | 15 | 0/18 | | |
| 26 | 73 | + | 23 | D | 2 | – | 11/11 | Bones | |
| 27 | 40 | – | 22 | U | 2 | 35 | 4/17 | | |
| 28 | 49 | – | 18 | D | 3 | 30 | 2/11 | | |
| 29 | 43 | – | 19 | L | 2 | 20 | 3/19 | | |
| 30 | 39 | – | 19 | U | 2 | 20 | 1/11 | | |
| 31 | 51 | + | 24 | D | 2 | 15 | 3/ 8 | | |
| 32 | 77 | + | 27 | D | 3 | 10 | 1/15 | | |

[a]Body mass index ($W/H^2$). [b]D, invasive ductal carcinoma; L, invasive lobular carcinoma; U, invasive carcinoma of uncertain type. [c]Grading according to Bloom & Richardson (1957). [d]mm, minimal size measured on the carcinoma during pathological examination. [e]Number of positive (L) lymph-nodes/examined (E) lymph-nodes.

**Table II** Fatty acid composition of breast tumour phosphatidylethanolamine

| | Phosphatidylethanolamine | |
|---|---|---|
| *Fatty acids* | *Mean (%)*[a] | *Range (%)* |
| Saturated | | |
| 16:0[b] | 5.8 | 1.2–10.4 |
| 18:0 | 18.7 | 9.9–33.3 |
| Unsaturated | | |
| 16:1 ω7 | 1.4 | 0.1–5.9 |
| 18:1[c] | 23.5 | 12.0–39.9 |
| 18:2 ω6 | 9.3 | 2.8–16.6 |
| 20:2 ω6 | 0.4 | 0.1–5.2 |
| 20:3 ω6 | 2.1 | 0.4–9.6 |
| 20:4 ω6 | 20.1 | 8.8–43.7 |
| 22:6 ω3 | 3.5 | 0.3–6.8 |
| DMA[d] | 5.7 | 0.1–15.4 |

[a]Expressed as % of total area; variation among measures was less than 2%; non identified fatty acids accounted for less than 4.8%. [b]Denoted as number of carbons: number of double bonds. [c]Expressed as the sum of 18:1 ω7 + 18:1 ω9. [d]Dimethyl acetals of fatty aldehydes.

**Figure 1** Distribution of patients by their intra-tumour membrane level of n-6 polyunsaturated fatty acids in phosphatidylethanolamine. Hatched frames represent patients without relapses and closed frames patients in whom metastases occurred during the follow-up period. Lipids were prepared from the primary tumor and separated in phospholipid classes as described in methods. Fatty acids composition of phosphatidylethanolamine (PE) was determined by gas chromatography. n-6 PUFA refers to the sum of linoleic and arachidonic acids, expressed as % of the total fatty acid content of PE.

patients with very different specific dietary habits among a group with an identical culture and living in the same area seems unlikely. In any case, the modalities of tumour specimens collection used in the present study could not have allowed such a selection.

No data relating dietary fat to tumour membrane fatty acid composition are available in humans. However, epidemiological studies suggest that the quantity and the quality of dietary fat intake are associated with differences in post-treatment survival rates (Wynder *et al.*, 1986; Gregorio *et al.*, 1985). Another study found an effect of body weight, but not of dietary fat, on survival (Newman *et al.*, 1986). A relationship was recently reported between dietary intake of polyunsaturated fatty acids before diagnosis and the lack of axillary lymph node extension of tumours (Verreault *et al.*, 1988), a clinical situation associated with a lower probability of subsequent metastasis. It is therefore possible that the quality of dietary fat influences not only tumour incidence, but also breast cancer presentation.

**Figure 2** Probability of remaining metastasis-free according to the n-6 PUFA contents of tumour phosphatidylethanolamine. Continuous line and dashed line represent patients with levels of n-6 PUFA respectively above or below 28% of total fatty acids. Proportion of N + patients: 8/14 and 10/18. $\chi^2 = 6.21$, $P<0.02$.

**Presently, the clinical management of breast cancer patients relies on each patient's individual risk factors (McGuire, 1989). The low level of n-6 PUFA in the primary tumour appears to be a predictor of subsequent metastasis, although its independence from other prognostic factors should be thoroughly evaluated in an expanded study.**

We thank F. Gauthier, F. Barin, L. Emorine and N. Salem Jr for helpful discussion; F. Fetissoff for comparatively reappraising the histopathology of the tumours. This work was supported by grants from the Ligue Nationale Française Contre le Cancer (LNFCC, Comités National et Départmental du Cher); P. Besson was a recipient of a LNFCC fellowship (Comité du Cher).

### References

BLOOM, H.J.G. & RICHARDSON, W.W. (1957). Histologic grading and prognosis in breast cancer. *Br. J. Cancer*, **11**, 359.

BOUGNOUX, P., SALEM, N., LYONS, C. & HOFFMAN, T. (1985). Alteration in the membrane fatty acid composition of human lymphocytes and cultured transformed cells induced by interferon. *Mol. Immunol.*, **22**, 1107.

COX, D.R. (1972). Regression models and life tables (with discussion). *J.R. Stat. Soc.*, **34**, 197.

GINSBERG, B.H., BROWN, T.J., SIMON, I. & SPECTOR, A.A. (1981). Effect of the membrane lipid environment on the properties of insulin receptors. *Diabetes*, **30**, 773.

GREGORIO, D.I., EMRICH, L.J., GRAHAM, S., MARSHALL, J.R. & NEMOTO, T. (1985). Dietary fat consumption and survival among women with breast cancer. *J. Natl Cancer Inst.*, **75**, 37.

HARRIS, J.R., HELLMAN, S., CANELLOS, G.P. & FISHER, B. (1985). Cancer of the breast. In *Cancer, Principles and Practice of Oncology*, 2nd edn, De Vita, V.T. Jr, Hellman, S. & Rosenberg, S.A. (eds) p. 1119. Lippincott: Philadelphia.

HUBBARD, N.E. & ERICKSON, K.L. (1987). Enhancement of metastasis from a transplantable mouse mammary tumor by dietary linoleic acid. *Cancer Res.*, **47**, 6171.

KATZ, E.B. & BOYLAN, E.S. (1987). Stimulatory effect of high polyunsaturated fat diet on lung metastasis from the 13762 mammary adenocarcinoma in female retired breeder rats. *J. Natl Cancer Inst.*, **79**, 351.

MCGUIRE, W.L. (1989). Adjuvant therapy of node-negative breast cancer. *N. Engl. J. Med.*, **320**, 525.

NEWMAN, S.C., MILLER, A.B. & HOWE, G.R. (1986). A study of the effect of weight and dietary fat on breast cancer survival time. *Am. J. Epidemiol.*, **123**, 767.

NOWELL, P.C. (1986). Mechanisms of tumor progression. *Cancer Res.*, **46**, 2203.

SPECTOR, A.A. & YOREK, M.A. (1985). Membrane lipid composition and cellular function. *J. Lipid Res.*, **26**, 1015.

SPECTOR, A.A. & BURNS, C.P. (1987). Biological and therapeutic potential of membrane lipid modification in tumors. *Cancer Res.*, **47**, 4529.

STUBBS, C.D. & SMITH, A.D. (1984). The modification of mammalian membrane polyunsaturated fatty acid composition in relation to membrane fluidity and function. *Biochim. Biophys. Acta*, **779**, 89.

TINSLEY, I.J. (1989). Dietary fatty acids and mammary tumorigenesis. In *Carcinogenesis and Dietary Fat*, Abraham, S. (ed.) p. 101. Kluwer: Boston.

VERREAULT, R., BRISSON, J., DESCHENES, L., NAUD, F., MEYER, F. & BELANGER, L. (1988). Dietary fat in relation to prognostic indicators in breast cancer. *J. Natl Cancer Inst.*, **80**, 819.

WYNDER, E.L., ROSE, D.P. & COHEN, L.A. (1986). Diet and breast cancer in causation and therapy. *Cancer*, **58**, 1804.